# OHCCPredictor: an online risk stratification model for predicting survival duration of older patients with hepatocellular carcinoma

**DOI:** 10.1007/s12072-023-10516-x

**Published:** 2023-04-17

**Authors:** Juntao Tan, Yue Yu, Xiantian Lin, Yuxin He, Wen Jin, Hong Qian, Ying Li, Xiaomei Xu, Yuxi Zhao, Jianwen Ning, Zhengyu Zhang, Jingjing Chen, Xiaoxin Wu

**Affiliations:** 1 Operation Management Office, Affiliated Banan Hospital of Chongqing Medical University, Chongqing, 401320 China; 2 Senior Bioinformatician Department of Quantitative, Health Sciences Mayo Clinic, Rochester, MN 55905 US; 3 State Key Laboratory for Diagnosis and Treatment of Infectious Diseases, National Clinical Research Centre for Infectious Diseases, The First Affiliated Hospital, Zhejiang University School of Medicine, 79 Qing Chun Road, Hangzhou, Zhejiang, 310003 China; 4 Department of Medical Administration, Affiliated Banan Hospital of Chongqing Medical University, Chongqing, 401320 China; 5 Medical Records Department, The First Affiliated Hospital, Zhejiang University School of Medicine, Hangzhou, 310003 China; 6 Medical Records Department, The First Hospital of Lanzhou University, Lanzhou, 730000 China; 7 Department of Medical Administration, The First Affiliated Hospital, Zhejiang University School of Medicine, Hangzhou, 310003 China; 8 Department of Gastroenterology, Chengdu Fifth People’s Hospital, Chengdu, 611130 China; 9 Department of Infectious Diseases, The First Affiliated Hospital of Chongqing Medical University, Chongqing, 404000 China; 10 Emergency Department, The First Affiliated Hospital, Zhejiang University School of Medicine, Hangzhou, 310003 China; 11 Department of Digital Urban Governance, Zhejiang University City College, Hangzhou, 310015 China

**Keywords:** Hepatocellular carcinoma, Survival duration, Nomogram, Risk stratification, Predictive model

## Abstract

**Background:**

Although the elderly constitute more than a third of hepatocellular carcinoma (HCC) patients, they have not been adequately represented in treatment and prognosis studies. Thus, there is not enough evidence to guide the treatment of such patients. The objective of this study is to identify the prognostic factors of older patients with HCC and to construct a new prognostic model for predicting their overall survival (OS).

**Methods:**

2,721 HCC patients aged ≥ 65 were extracted from the public database-Surveillance, Epidemiology, and End Results (SEER) and randomly divided into a training set and an internal validation set with a ratio of 7:3. 101 patients diagnosed from 2008 to 2017 in the First Affiliated Hospital of Zhejiang University School of Medicine were identified as the external validation set. Univariate cox regression analyses and multivariate cox regression analyses were adopted to identify these independent prognostic factors. A predictive nomogram-based risk stratification model was proposed and evaluated using area under the receiver operating characteristic curve (AUC), calibration curves, and a decision curve analysis (DCA).

**Results:**

These attributes including age, sex, marital status, T stage, N stage, surgery, chemotherapy, tumor size, alpha-fetoprotein level, fibrosis score, bone metastasis, lung metastasis, and grade were the independent prognostic factors for older patients with HCC while predicting survival duration. We found that the nomogram provided a good assessment of OS at 1, 3, and 5 years in older patients with HCC (1-year OS: (training set: AUC = 0.823 (95%CI 0.803–0.845); internal validation set: AUC = 0.847 (95%CI 0.818–0.876); external validation set: AUC = 0.732 (95%CI 0.521–0.943)); 3-year OS: (training set: AUC = 0.813 (95%CI 0.790–0.837); internal validation set: AUC = 0.844 (95%CI 0.812–0.876); external validation set: AUC = 0.780 (95%CI 0.674–0.887)); 5-year OS: (training set: AUC = 0.839 (95%CI 0.806–0.872); internal validation set: AUC = 0.800 (95%CI 0.751–0.849); external validation set: AUC = 0.821 (95%CI 0.727–0.914)). The calibration curves showed that the nomogram was with strong calibration. The DCA indicated that the nomogram can be used as an effective tool in clinical practice. The risk stratification of all subgroups was statistically significant (*p* < 0.05). In the stratification analysis of surgery, larger resection (LR) achieved a better survival curve than local destruction (LD), but a worse one than segmental resection (SR) and liver transplantation (LT) (*p* < 0.0001). With the consideration of the friendship to clinicians, we further developed an online interface (OHCCPredictor) for such a predictive function (https://juntaotan.shinyapps.io/dynnomapp_hcc/). With such an easily obtained online tool, clinicians will be provided helpful assistance in formulating personalized therapy to assess the prognosis of older patients with HCC.

**Conclusions:**

Age, sex, marital status, T stage, N stage, surgery, chemotherapy, tumor size, AFP level, fibrosis score, bone metastasis, lung metastasis, and grade were independent prognostic factors for elderly patients with HCC. The constructed nomogram model based on the above factors could accurately predict the prognosis of such patients. Besides, the developed online web interface of the predictive model provide easily obtained access for clinicians.

**Supplementary Information:**

The online version contains supplementary material available at 10.1007/s12072-023-10516-x.

## Introduction

Primary liver cancer is the sixth most common malignant tumor and the fourth leading cause of cancer-related deaths worldwide [1, 2]. In 2020, 907,100 new cases of primary liver cancer were reported, and 8.3% of patients died of it [3]. Hepatocellular carcinoma (HCC) is the most common type of primary liver cancer [4]. It usually occurs in those patients who suffer from chronic inflammation and fibrosis caused by viral hepatitis, alcohol, and metabolic-related fatty liver disease [5]. HCC’s 5-year overall survival (OS) rate is often less than 20% [6]. Tumor resection is the most effective treatment for early stage HCC, but the recurrence and metastasis rates are high and the prognosis of patients with HCC is usually poor [7, 8].

Improvements in the treatment of chronic liver diseases and the extension of life expectancy have resulted in an increase in the number of elderly patients with HCC. Although the elderly constitute more than a third of HCC patients, they have not been adequately represented in treatment and prognosis studies. Thus, there is not enough evidence to guide the treatment of such patients [9, 10]. The incidence rate of HCC is estimated to increase by approximately 59% by 2030, whereas individuals aged 65 or above are expected to constitute over 50% HCC patient by then [11].

Nomogram is considered a widely used predictive model for evaluating the prognosis of cancer patients [12–14]. In this study, we aim to identify the prognostic factors of elderly patients with HCC and construct a new prognostic model for predicting their OS, which would facilitate the provision of therapy suggestions and assist clinical decision-making.

## Materials and methods

### Subject selection

The Surveillance, Epidemiology, and End Results (SEER) is a dominant cancer statistics database in the United States (US) [15]. The database contains the diagnosis, treatment, and survival data of millions of cancer patients in US and other countries. The data regarding HCC patients during 2010–2015 were selected and handled by SEER*Stat 8.3.9 (https://seer.cancer.gov/) in this study. The inclusion and exclusion criteria are outlined in Supplementary Fig. 1. To be included, the patients must match the criteria: (1) diagnosed with HCC between 2010 and 2015 and (2) 65 years or older. Contrarily, we excluded: (1) patients for whom liver cancer was not their first primary tumor, (2) those with unknown alpha-fetoprotein (AFP) level, (3) those without follow-up time and (4) those with missing survival data. Moreover, we retrospectively enrolled elderly patients diagnosed with HCC to construct the external validation set between 2008 and 2017 from the First Affiliated Hospital of Zhejiang University School of Medicine in light of the selection criteria. The protocol of this research was approved by the Ethics Committee of the First Affiliated Hospital of Zhejiang University School of Medicine (Ethical approval No. IIT20230048B).

### Variables selection

Fourteen variables were selected in this study: age at diagnosis, race, sex, marital status, T stage, N stage, surgery, radiotherapy, chemotherapy, tumor size, AFP level, bone metastasis, lung metastasis, and grade. Fibrosis score (FS) was also adopted in this study. Surgery was divided into five categories: no surgery, local destruction (LD), segmental resection (SR), larger resection (LR), and liver transplantation (LT). For LD patients, there exists a large range of therapeutic options including photodynamic therapy (PDT), electrocautery / fulguration (includes the use of hot forceps for tumor destruction), cryosurgery, laser, alcohol (percutaneous ethanol injection [PEI]), heat-radio-frequency ablation (RFA) and other methods (e.g., ultrasound, acetic acid). The above therapeutic options have been defined in SEER database. Considering that age and tumor size were continuous variables, X-tile was used to determine the optimal cutoff values for them [16]. The results showed that the best cutoff values for age were 74 and 80, whereas the best cutoff values for tumor size were 5.6 and 8.5 cm. OS was the outcome of the model proposed in this study, which was defined as the time from randomization until death from any cause or the date of the last follow-up.

### Statistical analysis

Statistical analysis was performed using SPSS 22.0 and R (version 4.0.2, Vienna, Austria). Values were considered statistically significant at *p* < 0.05. Univariate cox regression analysis and multivariate cox regression analysis were adopted to identify independent prognostic factors. The risk ratio (HR) and 95% confidence interval (CI) were used to show the impact of the variables on the patients’ survival. A nomogram was constructed based on these independent prognostic factors. The discriminatory values of the models were evaluated based on the concordance index (C-index). The area under the curve (AUC) of the receiver operating characteristic (ROC) curve was used to evaluate the prognostic accuracy of the nomogram. Calibration curve was generated to evaluate the calibration of the nomogram. In addition, a decision curve analysis (DCA) was performed to demonstrate the clinical benefit of the nomogram [17]. To streamline the power calculation estimation, we produced PowerTools—an interactive open-source web application that was written in R using the Shiny framework (http://www.shinyapps.io/).

In addition, we developed a risk stratification model based on the total score of each patient, as calculated by the nomogram. Then, X-Tile was used to determine the best cutoff value to divide patients into low-risk, intermediate-risk, and high-risk groups. Kaplan–Meier curves and log-rank tests were used to analyze and compare the OS of patients in different subgroups.

## Results

### Patient characteristics

A total of 2,721 patients extracted from the SEER database were divided into a training set (*N* = 1904) and an internal validation set (*N* = 817). The chi-square test showed that there was no significant difference between the two sets (Table 1). In the training set, 58.4% (1112/1904), 27.2% (518/1,904), and 14.4% (274/1904) of the patients were aged < 74, 74–80, and > 80, respectively. In addition, 52.0% (990/1904), 21.8% (415/1904), and 26.2% (499/1904) of the patients’ tumor sizes were < 5.6 cm, 5.6–8.5 cm, and > 8.5 cm, respectively. Furthermore, 62.9% (1197/1904) of the patients had elevated AFP levels, whereas 37.1% (707/1,904) of them had normal AFP levels. Patient characteristics of the external validation set (*N* = 101) was listed in Supplementary Table 1.

**Table 1:** Baseline: clinicopathological characteristics of the subjects

Variables	Total (*N* = 2721)		Training set (*N* = 1904)		Validation set (*N* = 817)		*p*
	No. of patients (%)	Median OS (95% CI)	No. of patients (%)	Median OS (95% CI)	No. of patients (%)	Median OS (95% CI)	
Age							0.697
< 74	1577 (58.0)	18.0 (16.8–19.2)	1112 (58.4)	17.0 (15.6–18.4)	465 (56.9)	18.0 (15.8–20.2)	
74–80	753 (27.6)	16.0 (14.4–17.6)	518 (27.2)	15.0 (13.2–16.8)	235 (28.8)	19.0 (15.9–22.1)	
> 80	391 (14.4)	13.0 (11.3–14.7)	274 (14.4)	13.0 (11.0–15.0)	117 (14.3)	14.0 (10.6–17.4)	
Race							0.578
Black	269 (9.9)	15.0 (12.6–17.4)	182 (9.5)	15.5 (12.6–18.4)	87 (10.6)	15.0 (10.7–19.3)	
White	1813 (66.6)	16.0 (15.0–17.0)	1279 (67.2)	15.0 (13.9–16.1)	534 (65.4)	18.0 (16.1–19.9)	
Others Δ	639 (23.5)	19.0 (17.0–21.0)	443 (23.3)	19.0 (16.7–21.3)	196 (24)	20.0 (16.3–23.7)	
Sex							0.320
Female	790 (29.0)	17.0 (15.4–18.6)	542 (28.5)	18.0 (16.0–20.0)	248 (30.4)	16.0 (13.2–18.8)	
Male	1,931 (71.0)	16.0 (15.0–17.0)	1,362 (71.5)	15.0 (13.9–16.1)	569 (69.6)	18.0 (16.0–20.0)	
Marriage							0.289
Married	1654 (60.8)	17.0 (15.9–18.1)	1,145 (60.1)	17.0 (15.7–18.3)	509 (62.3)	19.0 (16.8–21.2)	
Unmarried	1067 (39.2)	15.0 (13.8–16.2)	759 (39.9)	15.0 (13.6–16.4)	308 (37.7)	15.0 (12.8–17.2)	
T stage							0.301
T1	1,359 (49.9)	20.0 (18.6–21.4)	930 (48.8)	20.0 (18.4–21.6)	429 (52.5)	21.0 (18.5–23.5)	
T2	575 (21.1)	20.0 (17.9–22.1)	407 (21.4)	20.0 (17.6–22.4)	168 (20.6)	22.0 (17.9–26.1)	
T3	674 (24.8)	8.0 (7.0–9.0)	483 (25.4)	8.0 (6.8–9.2)	191 (23.4)	9.0 (6.9–11.1)	
T4	113 (4.2)	8.0 (5.3–10.7)	84 (4.4)	6.0 (3.2–8.8)	29 (3.5)	11.0 (4.1–17.9)	
*N* stage							0.429
N0	2577 (94.7)	17.0 (16.1–17.9)	1799 (94.5)	17.0 (16.0–18.0)	778 (95.2)	18.5 (16.8–20.2)	
N1	144 (5.3)	5.0 (3.6–6.4)	105 (5.5)	5.0 (3.3–6.7)	39 (4.8)	5.0 (2.4–7.6)	
Surgery							0.171
No	1472 (54.1)	10.0 (9.2–10.8)	1053 (55.3)	10.0 (9.1–10.9)	419 (51.2)	10.0 (8.5–11.5)	
LD	335 (12.3)	25.0 (22.9–27.1)	218 (11.4)	23.5 (21.0–26.0)	117 (14.3)	26.0 (22.4–29.6)	
SR	431 (15.8)	28.0 (26.0–30.0)	304 (16.0)	27.5 (25.1–29.9)	127 (15.5)	29.0 (25.2–32.8)	
LR	359 (13.2)	25.0 (22.9–27.1)	244 (12.8)	23.0 (20.5–25.5)	115 (14.1)	28.0 (24.0–32.0)	
LT	124 (4.6)	34.0 (30.4–37.6)	85 (4.5)	31.0 (26.2–35.8)	39 (4.8)	35.0 (29.8–40.2)	
Radiation							0.163
Yes	44 (1.6)	18.5 (10.6–26.4)	35 (1.8)	19.0 (9.7–28.3)	9 (1.1)	15.0 (0.5–29.5)	
No	2677 (98.4)	16.0 (15.2–16.8)	1869 (98.2)	16.0 (15.0–17.0)	808 (98.9)	18.0 (16.4–19.6)	
Chemotherapy							0.103
Yes	1076 (39.5)	15.0 (13.8–16.2)	772 (40.5)	15.0 (13.6–16.4)	304 (37.2)	16.0 (13.6–18.4)	
No	1645 (60.5)	17.0 (15.9–18.1)	1132 (59.5)	17.0 (15.7–18.3)	513 (62.8)	19.0 (16.9–21.1)	
Tumor size							0.231
< 5.6 cm	1444 (53.1)	21.0 (19.6–22.4)	990 (52)	21.0 (19.4–22.6)	454 (55.6)	23.0 (20.4–25.6)	
5.6–8.5 cm	580 (21.3)	14.0 (12.4–15.6)	415 (21.8)	14.0 (12.1–15.9)	165 (20.2)	14.0 (10.9–17.1)	
> 8.5 cm	697 (25.6)	9.0 (7.9–10.1)	499 (26.2)	8.0 (6.7–9.3)	198 (24.2)	9.0 (6.8–11.2)	
AFP							0.239
Normal	991 (36.4)	21.0 (19.4–22.6)	707 (37.1)	20.0 (18.1–21.9)	284 (34.8)	22.5 (19.3–25.7)	
Elevated	1730 (63.6)	14.0 (13.1–14.9)	1197 (62.9)	14.0 (12.9–15.1)	533 (65.2)	14.0 (12.2–15.8)	
FS							0.424
None to Moderate (0–4)	305	22 (19.7–24.3)	205	20.0 (17.2–22.8)	100	28.0 (23.7–32.3)	
Severe Fibrosis to Cirrhosis (5–6)	551	20.0 (18.4–21.6)	381	19.0 (17.0–21.0)	170	21.5 (18.7–24.3)	
Unknown	1865	15.0 (14.1–15.9)	1318	14.5 (13.5–15.5)	547	15.0 (13.3–16.7)	
Bone metastasis							0.061
No	2671 (98.2)	17.0 (16.1–17.9)	1863 (97.8)	16.0 (15.0–17.0)	808 (98.9)	18.0 (16.4–19.6)	
Yes	50 (1.8)	9.0 (6.6–11.4)	41 (2.2)	9.0 (6.3–11.7)	9 (1.1)	/	
Lung metastasis							0.103
No	2631 (96.7)	17.0 (16.1–17.9)	1,848 (97.1)	16.0 (15.0–17.0)	783 (95.8)	19.0 (17.3–20.7)	
Yes	90 (3.3)	3.5 (2.2–4.8)	56 (2.9)	4.0 (2.2–5.8)	34 (4.2)	2.5 (0.8–4.2)	
Grade							0.805
I	854 (31.4)	17.0 (15.5–18.5)	587 (30.8)	17.0 (15.2–18.8)	267 (32.7)	18.0 (15.3–20.7)	
II	1300 (47.8)	18.0 (16.7–19.3)	918 (48.2)	18.0 (16.5–19.5)	382 (46.8)	19.0 (16.6–21.4)	
III	531 (19.5)	11.0 (9.4–12.6)	373 (19.6)	10.0 (8.3–11.7)	158 (19.3)	14.0 (10.5–17.5)	
IV	36 (1.3)	8.0 (1.9–14.1)	26 (1.4)	10.5 (3.5–17.5)	10 (1.2)	/	

Δ Others include American Indian, AK Native, Asian and Pacific Islander; *LD* local destruction, *SR* segmental resection, *LR* larger resection, *LT* liver transplantation, *AFP* alpha fetoprotein, *FS* fibrosis score, *OS* overall survival, *CI* confidence interval

### Univariate and multivariate analysis for prognosis

**Fig. 1:**
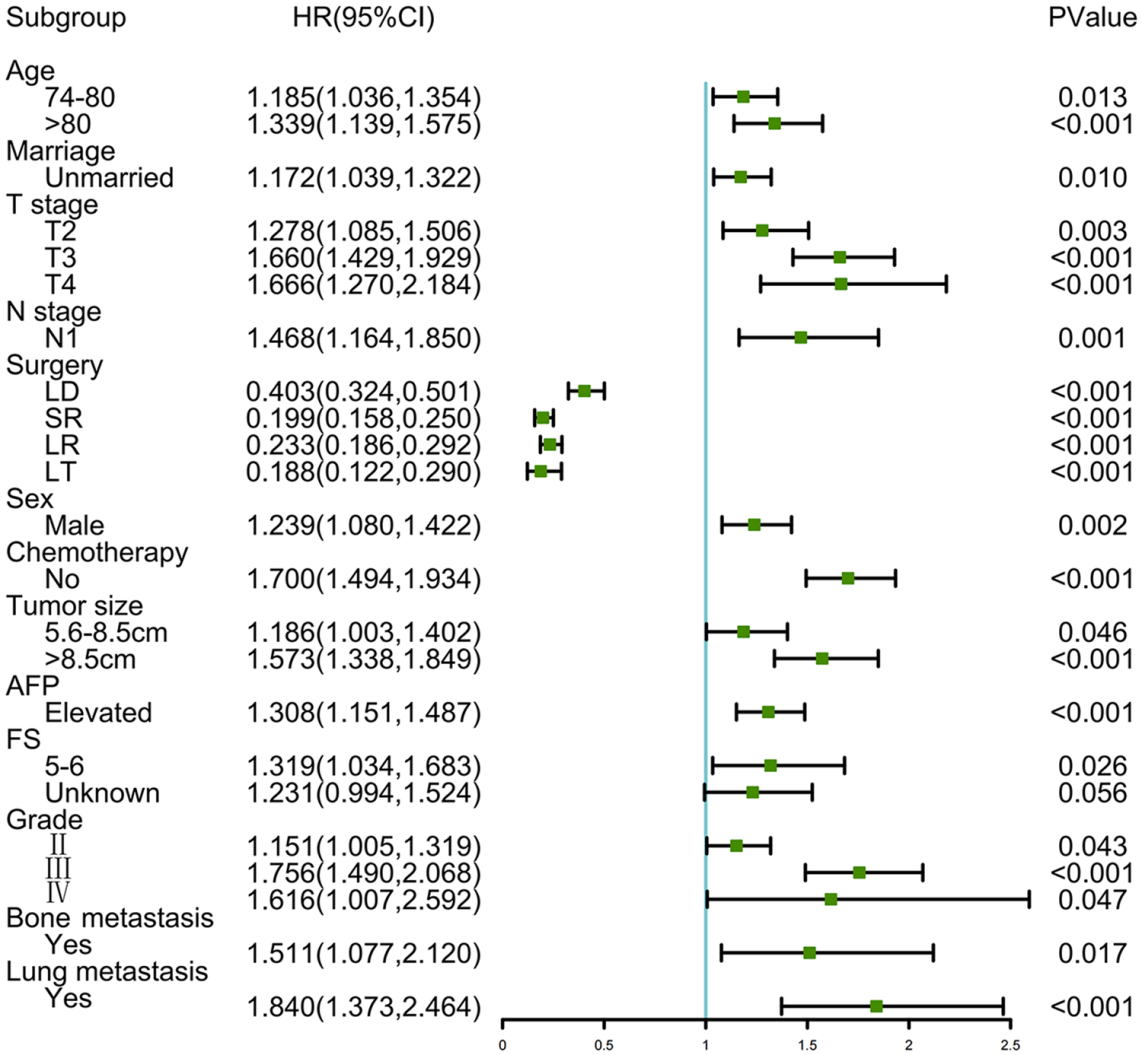
Forest plot showing the results of multivariate analysis for overall survival

To identify OS-related variables, 15 variables were used in the univariate cox analysis. The univariate cox regression analysis showed that all variables were statistically significant (*p* < 0.05). The multivariate cox analysis indicated that higher age, sex (male), being unmarried, higher T stage, higher N stage, no surgery, no chemotherapy, larger tumor size, elevated AFP, higher fibrosis score, bone metastasis, lung metastasis, and higher grade were all independently associated with poor OS of HCC patients, as shown in Table 2 and Fig. 1.

**Table 2:** Univariate and multivariate analyses for overall survival of the subjects

Variables	Univariate Cox			Multivariate Cox		
	HR	95%CI	*p*	HR	95%CI	*p*
Age						
< 74	Reference			Reference		
74–80	1.280	1.124–1.459	< 0.001	1.184	1.036–1.354	0.014
> 80	1.784	1.531–2.080	< 0.001	1.323	1.125–1.556	< 0.001
Race						
Black	Reference			Reference		
White	0.966	0.797–1.170	0.721	1.175	0.963–1.433	0.112
Other	0.729	0.586–0.908	0.005	0.934	0.742–1.174	0.559
Sex						
Female	Reference			Reference		
Male	1.201	1.057–1.365	0.005	1.211	1.055–1.391	0.007
Marriage						
Married	Reference			Reference		
Unmarried	1.228	1.096–1.377	< 0.001	1.160	1.026–1.312	0.018
T stage						
T1	Reference			Reference		
T2	1.099	0.941–1.283	0.232	1.271	1.079–1.498	0.004
T3	2.625	2.230–3.000	< 0.001	1.636	1.408–1.900	< 0.001
T4	2.814	2.197–3.606	< 0.001	1.636	1.247–2.147	< 0.001
*N* stage						
N0	Reference			Reference		
N1	2.832	2.287–3.506	< 0.001	1.449	1.150–1.826	0.002
Surgery						
No	Reference			Reference		
LD	0.353	0.289–0.430	< 0.001	0.417	0.335–0.520	< 0.001
SR	0.214	0.174–0.263	< 0.001	0.208	0.165–0.262	< 0.001
LR	0.311	0.254–0.381	< 0.001	0.245	0.196–0.307	< 0.001
LT	0.156	0.103–0.236	< 0.001	0.183	0.118–0.283	< 0.001
Radiation						
Yes	Reference			Reference		
No	1.720	1.050–2.818	0.031	0.987	0.596–1.636	0.960
Chemotherapy						
Yes	Reference			Reference		
No	0.786	0.701–0.881	< 0.001	1.655	1.455–1.882	< 0.001
Tumor size						
< 5.6 cm	Reference			Reference		
5.6–8.5 cm	1.639	1.418–1.894	< 0.001	1.178	0.996–1.392	0.056
> 8.5 cm	2.393	2.098–2.730	< 0.001	1.561	1.328–1.836	< 0.001
AFP						
Normal	Reference			Reference		
Elevated	1.517	1.344–1.711	< 0.001	1.320	1.161–1.500	< 0.001
FS						
None to Moderate (0–4)	Reference			Reference		
Severe Fibrosis to Cirrhosis (5–6)	1.239	0.977–1.570	0.076	1.314	1.029–1.677	0.028
Unknown	1.697	1.379–2.090	< 0.001	1.233	0.997–1.526	0.054
Bone metastasis						
No	Reference			Reference		
Yes	2.697	1.948–3.734	< 0.001	1.496	1.066–2.099	0.020
Lung metastasis						
No	Reference			Reference		
Yes	3.835	2.902–5.069	< 0.001	1.749	1.307–2.340	< 0.001
Grade						
I	Reference			Reference		
II	0.898	0.787–1.025	0.112	1.159	1.011–1.328	0.034
III	1.484	1.270–1.735	< 0.001	1.736	1.473–2.046	< 0.001
IV	1.393	0.878–2.209	0.159	1.574	0.981–2.525	0.060

*LD* local destruction, *SR* segmental resection, *LR* larger resection, *LT* liver transplantation, *AFP* alpha fetoprotein, *FS* Fibrosis score, *HR* hazard ratio, Overall Survival, *CI* confidence interval

### Prognostic nomogram model

**Fig. 2:**
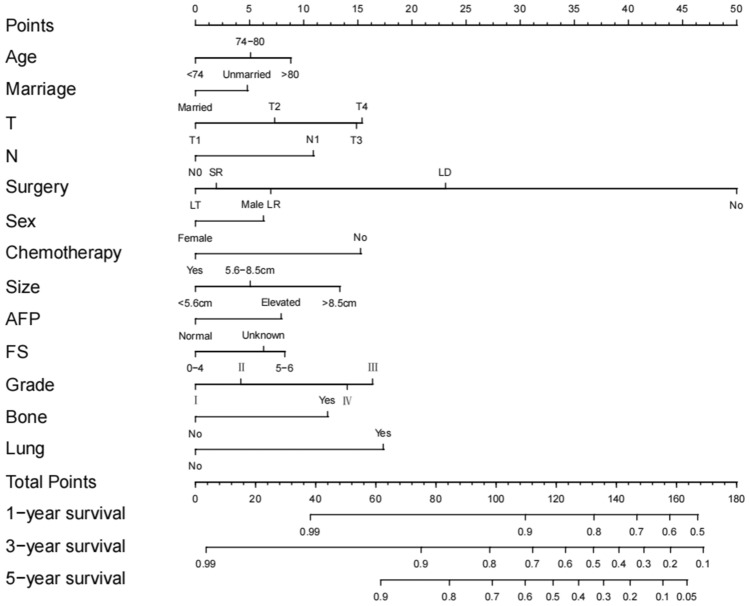
Nomogram for predicting 1-year, 3-year and 5-year overall survival of older HCC patients

**Fig. 3:**
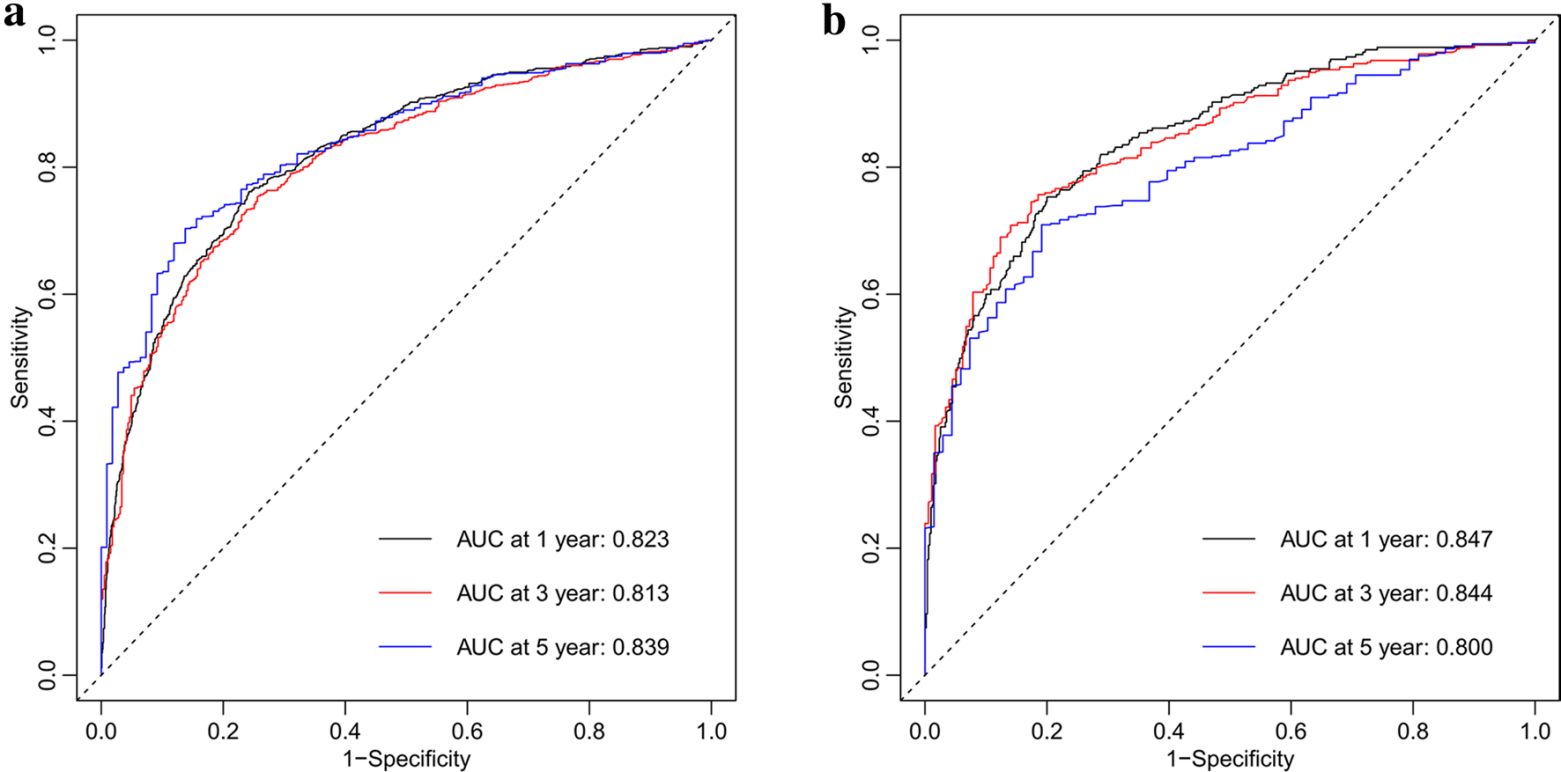
Receiver operating characteristic (ROC) curves of nomogram in training set **A** & internal validation set **B**

A nomogram was constructed based on all independent OS-related factors from the training set (as shown in Fig. 2), and then scores were assigned to the clinical variables in each subgroup (as shown in Table 3). Meanwhile, the time-dependent ROC curves showed that the AUC values at 1, 3, and 5 years were 0.823(95%CI 0.803–0.845), 0.813 (95%CI 0.790–0.837), and 0.839 (95%CI 0.806–0.872), respectively. This suggested favorable discrimination of the nomogram (as shown in Fig. 3). In the internal validation set, the AUC values at 1, 3, and 5 years were 0.847 (95%CI 0.818–0.876), 0.844 (95%CI 0.812–0.876), and 0.800 (95%CI 0.751–0.849), respectively. In the external validation set, the AUC values at 1, 3, and 5 years were 0.732 (95%CI 0.521–0.943), 0.780 (95%CI 0.674–0.887), and 0.821 (95%CI 0.727–0.914), respectively (as shown in Supplementary Fig. 2).

**Table 3:** Scores of clinical variables in each subgroup

Variables	Points	Variables	Points
Age		Chemotherapy	
< 74	0	Yes	0
74–80	5	No	15
> 80	9	Tumor size	
Sex		< 5.6 cm	0
Female	0	5.6–8.5 cm	5
Male	6	> 8.5 cm	13
Marriage		AFP	
Married	0	Normal	0
Unmarried	5	Elevated	8
*T* stage		FS	
T1	0	None to Moderate (0–4)	0
T2	7	Severe Fibrosis to Cirrhosis (5–6)	8
T3	15	Unknown	6
T4	15	Bone metastasis	
*N* stage		No	0
N0	0	Yes	12
N1	11	Lung metastasis	
Surgery		No	0
LT	0	Yes	17
SR	2	Grade	
LR	7	I	0
LD	23	II	4
No	50	III	16
		IV	14

*LD* local destruction, *SR* segmental resection, *LR* larger resection, *LT* liver transplantation, *AFP* alpha fetoprotein, *FS* Fibrosis score

**Fig. 4:**
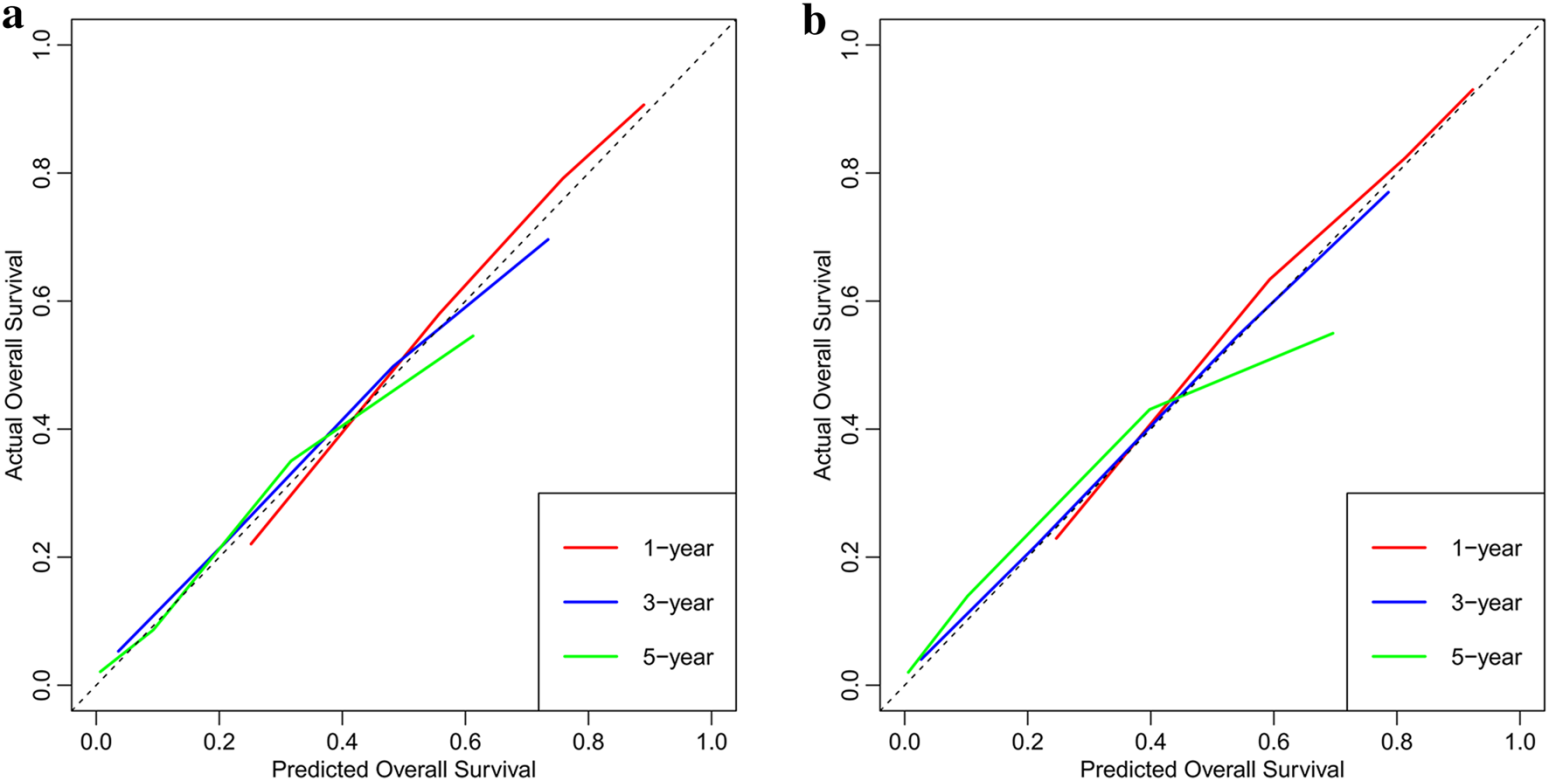
Calibration curves of nomogram at 1 year, 3 years, and 5 years for training set **A** and internal validation set **B**

**Fig. 5:**
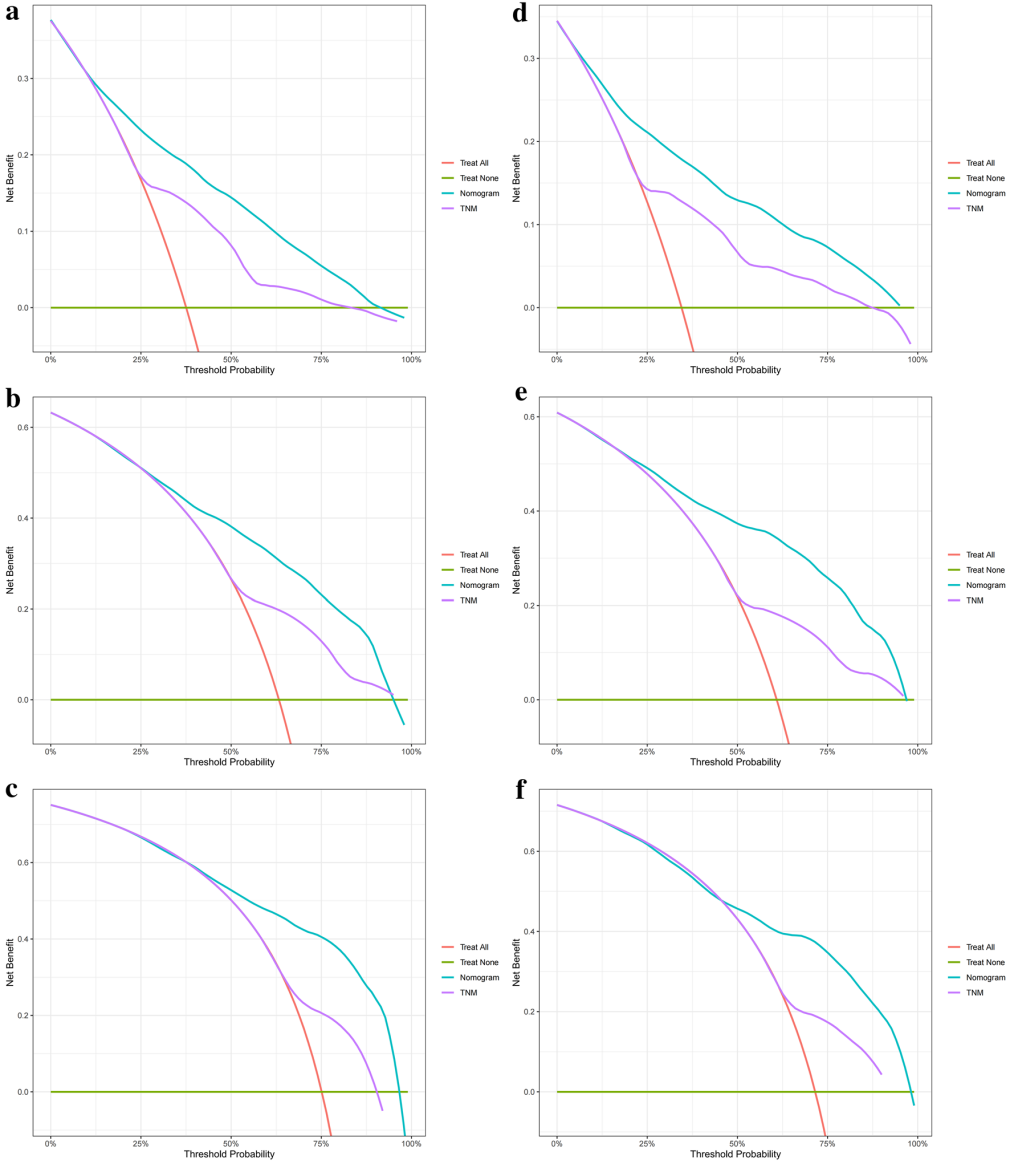
Decision curve analysis at 1 year, 3 years, and 5 years for training set **A–C** and internal validation set **D–F**

Moreover, the calibration curves (bootstraps = 1000) of the training set and internal validation set indicated that the nomogram had a strong calibration (as shown in Fig. 4). In addition, we compared the clinical practicability of the nomogram and the TNM staging system using DCA. The results indicated that the nomogram had a better clinical benefit and a larger threshold probability range, which confirmed that the nomogram can be used as an effective tool in clinical practice (as shown in Fig. 5).

### Risk stratification model

**Fig. 6:**
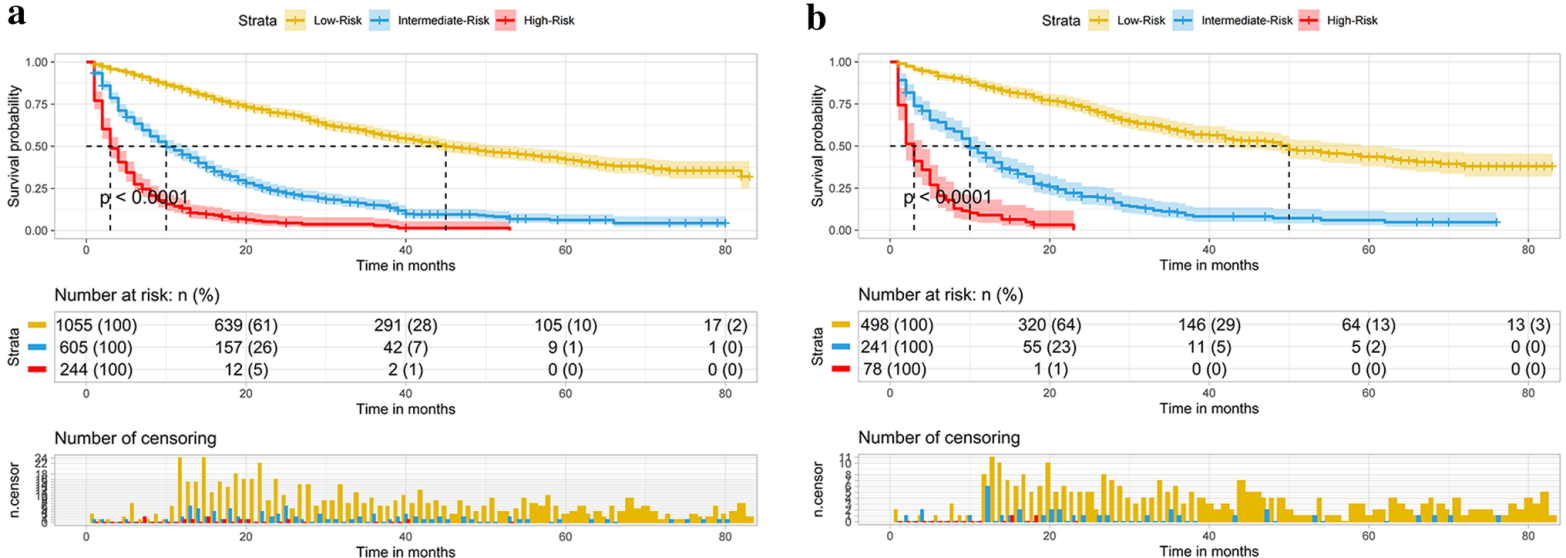
Survival curves showed the survival status classified by our nomogram of the training set **A** and internal validation **B** in HCC patients

Based on the constructed prognostic nomogram model, the patients were divided into the low-risk group (1,553/2,721, 57.1%; total score < 83), intermediate-risk group (846/2,721, 31.1%; total score 83–113), and high-risk group (322/2,721, 11.8%; total score > 113). The results of the Kaplan–Meier survival analysis with log-rank tests showed that there were different survival patterns among patients in the three groups. The prognosis of patients in the low-risk group was significantly better than that of those in the high-risk group (*p* < 0.0001) (as shown in Fig. 6).

### Risk stratification for subgroup analysis

**Fig. 7:**
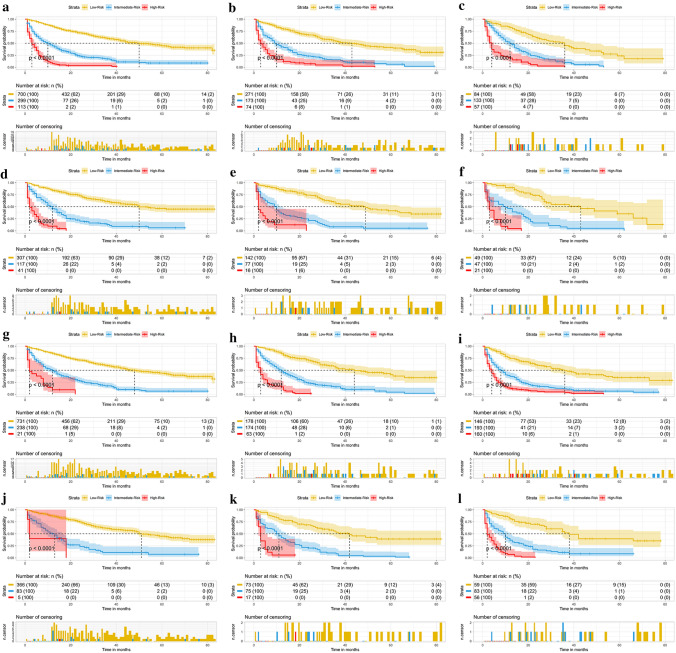
Subgroup analysis of aged < 74, 74–80, and > 80 in training set **A–C** and internal validation set **D–F** Subgroup analysis of tumor size < 5.6 cm, 5.6–8.5 cm, and > 8.5 cm in training set **G–I** and internal validation set **J–L**

**Fig. 8:**
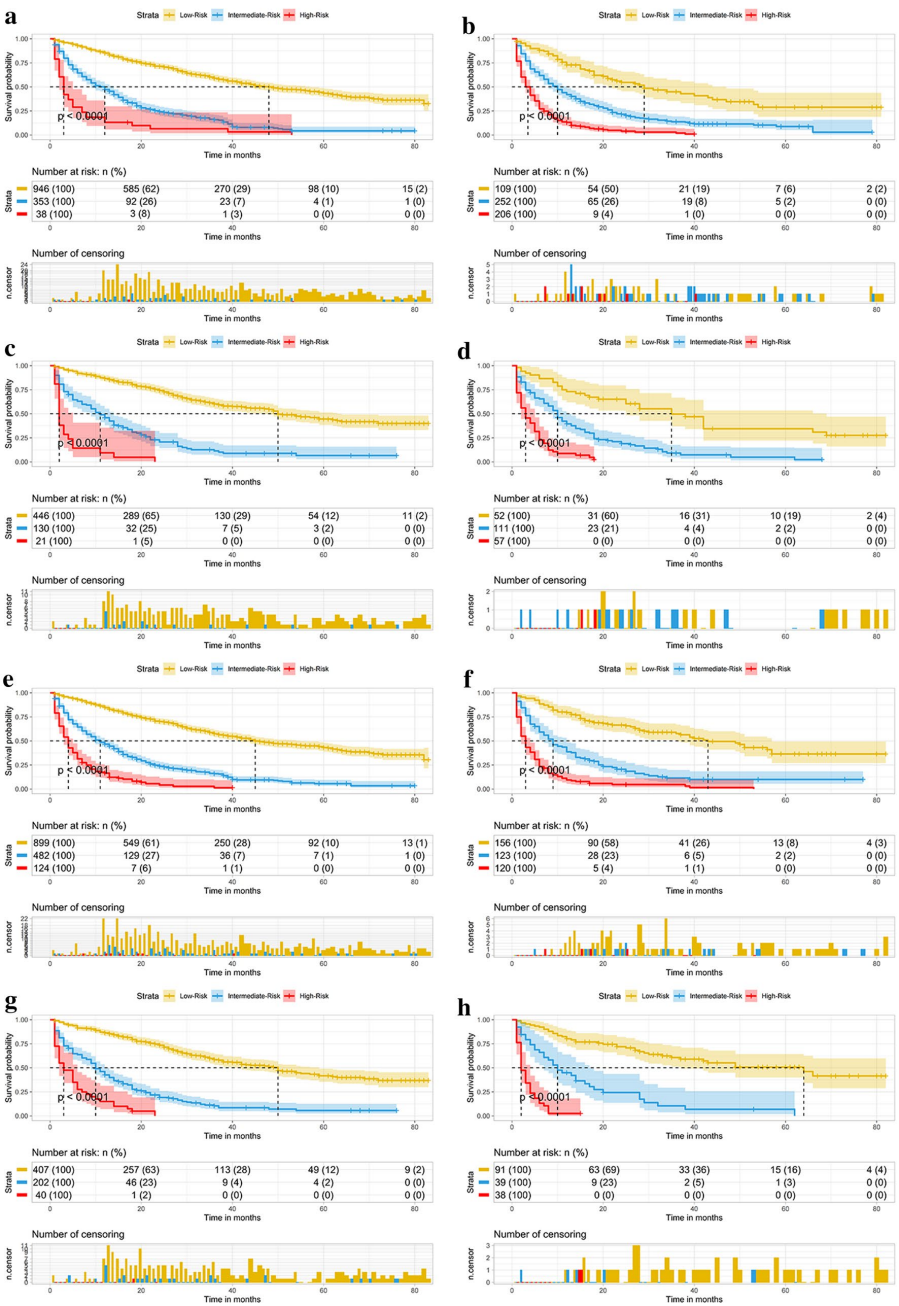
Subgroup analysis of T1–2 and T3–4 in training set **A**, **B** and internal validation set **C**, **D**; Subgroup analysis of grade I–II and grade III–IV in training set **E**, **F** and internal validation set **G**, **H**

**Fig. 9:**
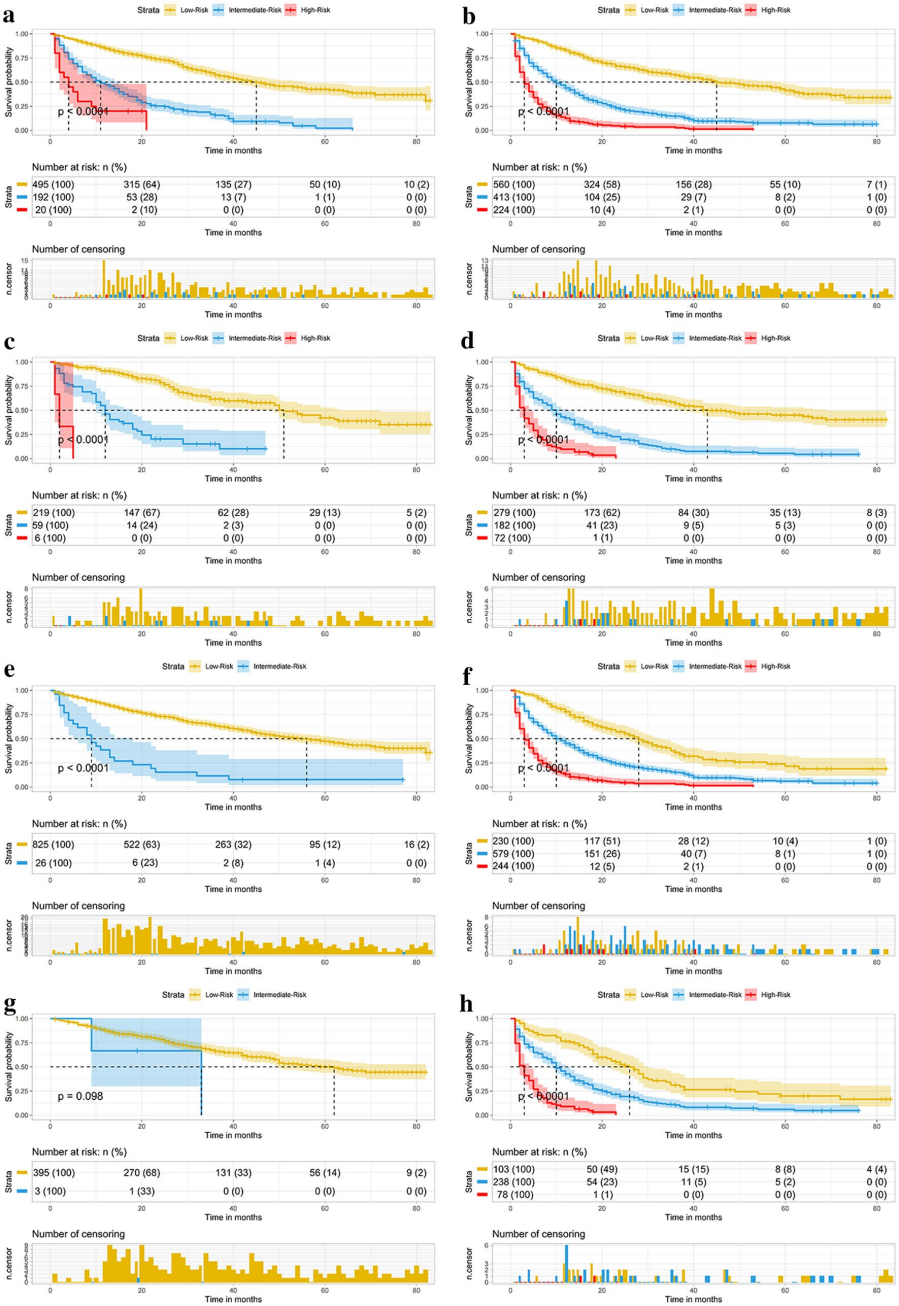
Subgroup analysis of normal AFP and elevated AFP in training set **A**, **B** and internal validation set **C**, **D** Subgroup analysis of surgery and no surgery in training set **E**, **F** and internal validation set **G**, **H**

Although the constructed nomogram model worked well in the training and internal validations, its effectiveness in the subgroups was unclear. Therefore, we divided these patients into different subgroups according to the age, tumor size, T stage, grade, AFP level and surgery, to further confirm the effectiveness of the nomogram. The results indicated that in both the training and internal validations, the risk stratification of all subgroups was statistically significant (*p* < 0.05). This implied that the nomogram was effective for the distinction of the prognosis of different subgroups of HCC patients **(**as shown in Figs. 7, 8, and 9).

### Stratification analysis of surgery

To further determine whether more radical surgeries should be taken for the older patients, we also performed surgical stratification analysis based on the univariate OS Cox model (age, T stage, tumor size, fibrosis score and grade). The results showed that LT had the best survival duration in the < 74 age band: HR 0.165 (95%CI 0.116–0.236), and unexpectedly, LR did not show better survival duration than SR in all ages ranges [< 74: HR 0.257 (95%CI 0.202–0.328) vs. HR 0.191 (95%CI 0.151–0.241); 74–80: HR 0.316 (95%CI 0.237–0.423) vs. HR 0.244 (95%CI 0.178–0.335); > 80: HR 0.322 (95%CI 0.205–0.507) vs. HR 0.169 (95%CI 0.092–0.312)]. Meanwhile, SR had the best survival duration in the 0–4 FS band: HR 0.215 (95%CI 0.132–0.351), LT had the best survival duration in the 5–6 FS band: HR 0.126 (95%CI 0.073–0.218) (as shown in Table 4).

**Table 4:** Stratification analysis of surgery

Variables	Non-surgery	Local destruction	Segmental resection	Larger resection	Liver transplantation
	N/reference	N/HR (95%CI)	N/HR (95%CI)	N/HR (95%CI)	N/HR (95%CI)
Age					
< 74	779	189	284	205	120
	1	0.323 (0.257–0.404)	0.191 (0.151–0.241)	0.257 (0.202–0.328)	0.165 (0.116–0.236)
74–80	412	101	116	120	4
	1	0.379 (0.286–0.503)	0.244 (0.178–0.335)	0.316 (0.237–0.423)	/
> 80	281	45	31	34	0
	1	0.364 (0.244–0.545)	0.169 (0.092–0.312)	0.322 (0.205–0.507)	/
*T* stage					
T1	603	230	292	181	53
	1	0.409 (0.333–0.503)	0.187 (0.146–0.239)	0.231 (0.175–0.306)	0.197 (0.115–0.335)
T2	253	83	92	83	64
	1	0.355 (0.251–0.504)	0.345 (0.245–0.485)	0.343 (0.240–0.491)	0.169 (0.103–0.279)
T3	539	21	32	76	6
	1	0.493 (0.299–0.812)	0.211 (0.121–0.367)	0.340 (0.247–0.467)	0.067 (0.009–0.475)
T4	77	1	15	19	1
	1	/	0.266 (0.130–0.542)	0.440 (0.248–0.780)	/
Tumor size					
< 5.6 cm	594	296	305	131	118
	1	0.430 (0.356–0.521)	0.237 (0.188–0.298)	0.270 (0.196–0.372)	0.196 (0.136–0.283)
5.6–8.5 cm	383	23	78	91	5
	1	0.409 (0.243–0.688)	0.207 (0.141–0.302)	0.211 (0.146–0.304)	0.138 (0.019–0.985)
> 8.5 cm	495	16	48	137	1
	1	0.501 (0.282–0.890)	0.211 (0.133–0.335)	0.292 (0.228–0.375)	/
FS					
None to Moderate (0–4)	88	21	85	103	8
	1	0.379 (0.203–0.709)	0.215 (0.132–0.351)	0.363 (0.245–0.539)	0.293 (0.105–0.814)
Severe Fibrosis to Cirrhosis (5–6)	244	100	96	42	69
	1	0.397 (0.294–0.538)	0.246 (0.171–0.353)	0.241 (0.140–0.415)	0.126 (0.073–0.218)
Unknown	1140	214	250	214	47
	1	0.326 (0.266–0.399)	0.190 (0.151–0.239)	0.283 (0.229–0.351)	0.166 (0.098–0.282)
Grade					
I	556	132	76	62	28
	1	0.305 (0.231–0.402)	0.178 (0.112–0.283)	0.262 (0.169–0.407)	0.132 (0.059–0.297)
II	611	160	254	192	83
	1	0.396 (0.315–0.497)	0.203 (0.160–0.257)	0.235 (0.182–0.304)	0.164 (0.107–0.252)
III	286	43	96	94	12
	1	0.300 (0.199–0.451)	0.162 (0.114–0.232)	0.287 (0.212–0.388)	0.077 (0.025–0.242)
IV	19	0	5	11	1
	1	/	0.074 (0.009–0.576)	0.272 (0.101–0.734)	/

*FS* fibrosis score, *HR* hazard ratio, *CI* confidence interval

Moreover, a more detailed plot was conducted on OS. It was shown that both in training set and internal validation, the surgery group had a higher survival curve than the non-surgery group did, and LT had the highest survival curve (*p* < 0.0001) (as shown in Supplementary Fig. 3). LR had a better survival curve than LD but a worse one than SR and LT (*p* < 0.0001).

### Online service of the constructed nomogram model

To provide easily obtained access of the proposed model, we developed an online website (https://juntaotan.shinyapps.io/dynnomapp_hcc/) to provide the service of nomogram model for medical staff. The provided function could automatically receive and calculate a patient’s survival probability. The scoring module enables the early identification of high-risk patients, which could further facilitate the appropriate treatment to prolong the survival time.

## Discussion

A total of 2,822 older patients with HCC were selected in this study. The univariate and multivariate cox regression analysis successfully identified 13 independent prognostic factors including age, sex, marital status, *T* stage, *N* stage, surgery, chemotherapy, tumor size, AFP level, fibrosis score, bone metastasis, lung metastasis, and grade. The prognostic nomogram model was constructed based on these factors. The C-index, calibration curve, and DCA evaluated the model based on discrimination, calibration, and clinical usefulness, respectively. In addition, we constructed a risk stratification model based on the total score of each patient provided by the nomogram.

In 2020, through univariate and multivariate Cox analyses, Liu and colleagues developed a full age spectrum prognosis model that included 6 predictors to evaluate the prognosis of HCC patients [18]. However, we considered that it is impossible to accurately evaluate the prognosis of elderly patients with HCC by constructing a full age spectrum prognosis model of patients with HCC, because the clinical and pathological characteristics of young and elderly patients with HCC are different. Previous studies have shown that it is necessary to build a prognostic model to precisely assessing of the prognosis of elderly patients with HCC [19, 20]. He et al. showed that age, race, T stage, histological grade, surgery, radiotherapy, and chemotherapy were independent predictors of cancer-specific survival in elderly patients with HCC [21]. Building upon their research, we have introduced two more important indicators (AFP level and fibrosis score) to evaluate the prognosis of elderly patients with HCC. The value of AFP level and fibrosis score in evaluating the prognosis of patients with HCC has been widely verified [22–24]. In addition, we also enrolled patients from other center to perform external validation of the prognostic model built in this study. In conclusion, this study has constructed a more accurate and applicable prediction model for evaluating the prognosis of elderly patients with HCC.

Being older, male, and unmarried, and with bone metastasis and lung metastasis were independently associated with poor OS of HCC patients. These results were consistent with those of previous research [25, 26]. Elderly patients usually suffer from comorbidities, such as malnutrition, decreased immune function, and cognitive impairment, which limit: their treatment options and thereby results in a worse prognosis [27, 28]. The results indicated that the older the patient, the shorter their OS and the worse their prognosis. Interestingly, we found that the prognosis of unmarried patients was worse than the married did. This may be caused by the limited ability of affording the costs of continued treatment, which forces them to shorten their treatment. In addition, single patients are unable to receive the emotional support that a spouse would provide, which adversely affects their prognosis. Bone metastasis is a typical metastatic pattern in patients with HCC. Literatures reported that incidence rate of bone metastasis of HCC patients ranged from 3 to 20%, and it was on an upward trend [29, 30]. Although the management of HCC patients was improved in recent years, the prognosis of those with bone metastasis remains weak. This study found that the prognosis of HCC patients with bone metastases was worse than the ones without bone metastases (HR: 1.511; 95%CI 1.077–2.120).

Tumor size, T stage, N stage, AFP level, fibrosis score and grade were proved as independent prognostic factors of HCC patients. AFP level and tumor size have been used in many HCC prognoses models and have been proven to have good predictive ability and evaluation effects [31–33]. AFP is a glycoprotein synthesized from embryonic liver cells. It is the first tumor marker discovered in HCC and is widely used for the diagnosis of HCC. For example, Bai et al. found that AFP-elevated was associated with inferior survival compared with AFP-normal in patients with HCC [34]. Wu et al. indicated that the survival rate of patients with HCC decreased with an increase in tumor size. The 5-year survival rate of patients with a tumor diameter of ≤ 2 cm was 21.9%, whereas the 5-year survival rate of patients with tumor sizes of 2.1–5.0, 5.1–10.0, and 10.1–20.0 cm decreased to 14.3%, 9.2%, and 7.7%, respectively [35]. The results in this study showed that compared to those with a tumor size of < 5.6 cm, the risk of HCC patients with tumor sizes of 5.6–8.5 cm and > 8.5 cm increased by 1.186 times and 1.573 times, respectively.

Surgery and adjuvant chemotherapy can significantly improve the prognosis of patients with HCC [36, 37]. With the development of medical technology, surgery has become a standard treatment for improving the prognosis of patients with HCC. Studies have shown that HCC patients with regional lymph node infiltration or multiple metastases may benefit from surgery [38, 39]. A cohort study reported that the median overall survival (OS) of patients treated with surgery was significantly longer than that of patients treated without surgery (MOS: 20 months vs. 7 months, *p *< 0.001) [40]. Another study provided evidence that adjuvant chemotherapy after hepatectomy was beneficial for patients with operable HCC [41]. Oxaliplatin, sorafenib, and 5-fluorouracil are common chemotherapeutic drugs. Two randomized, placebo-controlled phase III trials demonstrated a significant improvement in OS of patients with advanced HCC [42, 43]. A recent study demonstrated that hepatic artery infusion chemotherapy combined with sorafenib can improve the OS of patients with HCC [44]. The results in this study also showed that surgery and chemotherapy can significantly improve the prognosis of patients with HCC.

To reflect the clinical value of our research, we further performed a surgical stratification analysis of age, T stage, tumor size, fibrosis score and grade. In our study, all of the elderly patients with HCC treated with surgery, had longer survival duration than non-surgical patients, which was consistent with the previous studies [45, 46]. It was shown that SR was usually a better option for older patients with HCC over the age of 74 when LT was not available. Interestingly, we found that SR and LT were a better option for elderly patients with HCC in the 0–4 FS band and 5–6 FS band, respectively. To our knowledge, this finding was reported for the first time in the SEER database. It was also shown that both in training set and internal validation, LR did not show better survival than SR does, which was consistent with other findings [47]. When surgeons were faced with the dilemma of how to choose the best treatment solution for older patients with HCC who could not undergo LT, the finding in this study may provide valuable suggestion.

Nevertheless, this study has some limitations. First, this was a retrospective study, which might result in the introduction of a partial selection bias. Second, some potential prognostic factors such as ALBI grade for liver reserve, specific chemotherapy regimens and multigene signature assessment were not included in the SEER database. In addition, we lack information on clinical indicators other than AFP level and fibrosis score. More indicators included in the study could have helped to identify clinical indicators with high specificity and sensitivity in elderly HCC patients.

## Conclusion

In summary, age, sex, marital status, T stage, N stage, surgery, chemotherapy, tumor size, AFP level, fibrosis score, bone metastasis, lung metastasis, and grade were independent prognostic factors for older patients with HCC. The constructed nomogram model based on the above factors could accurately predict the prognosis of such patients. Besides, the developed online web interface of the predictive model provides easily obtained access for clinicians.

### Supplementary Information

Below is the link to the electronic supplementary material.

Supplementary file1 (DOCX 1397 KB)

## Data Availability

The raw data supporting the conclusions of this article will be made available by the authors, without undue reservation.
