# Supplementary material for: OHCCPredictor: an online risk stratification model for predicting survival duration of older patients with hepatocellular carcinoma
Source: Hepatol Int. 2023 Apr 17;18(2):550–67. doi: 10.1007/s12072-023-10516-x (PMC11014809; doi:10.1007/s12072-023-10516-x)
Supplement: Supplementary file 1 — Supplementary file1 (DOCX 1397 KB) [file 12072_2023_10516_MOESM1_ESM.docx]

**Supplementary information**

**Figure S1** Flow of inclusions and exclusions


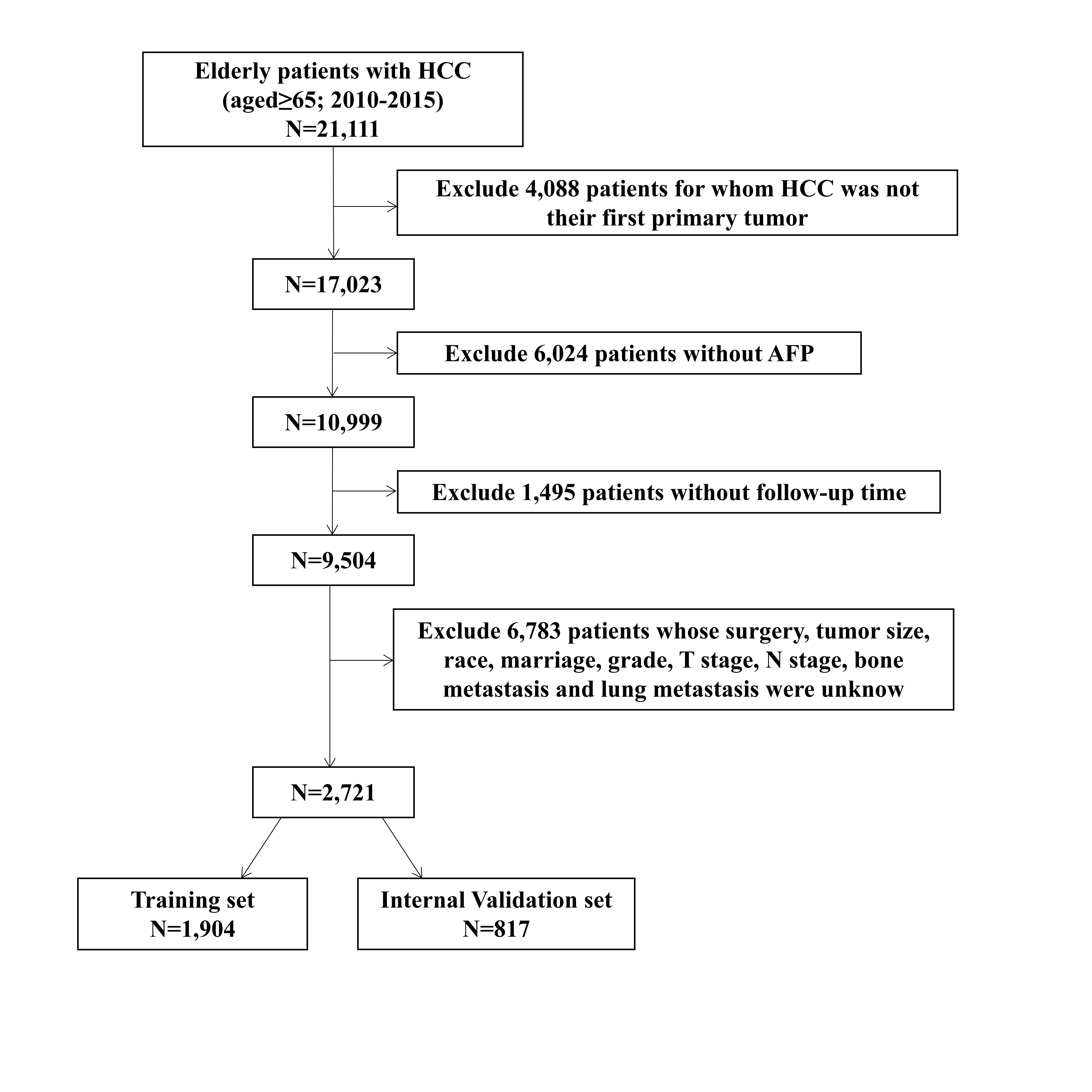


**Table S1** Patient characteristics of the external validation set

| Variables | No. of patients(%) |
| --- | --- |
| Age |  |
| <74 | 89(88.1) |
| 74-80 | 9(8.9) |
| >80 | 3(3.0) |
| Race |  |
| Black | 0(0.0) |
| White | 0(0.0) |
| Others Δ | 101(100.0) |
| Sex |  |
| Female | 21(20.8) |
| Male | 80(79.2) |
| Marriage |  |
| Married | 92(91.1) |
| Unmarried | 9(8.9) |
| T stage |  |
| T1 | 10(9.9) |
| T2 | 36(35.6) |
| T3 | 41(40.6) |
| T4 | 14(13.9) |
| N stage |  |
| N0 | 96(95.0) |
| N1 | 5(5.0) |
| Surgery |  |
| No | 5(5.0) |
| LD | 8(7.9) |
| SR | 59(58.4) |
| LR | 21(20.8) |
| LT | 8(7.9) |
| Radiation |  |
| Yes | 8(7.9) |
| No | 93(92.1) |
| Chemotherapy |  |
| Yes | 71(70.3) |
| No | 30(29.7) |
| Tumor size |  |
| <5.6cm | 75(74.2) |
| 5.6-8.5cm | 23(22.8) |
| >8.5cm | 3(3.0) |
| AFP |  |
| Normal | 62(61.4) |
| Elevated | 39(38.6) |
| FS |  |
| None to Moderate (0-4) | 19(18.8) |
| Severe Fibrosis to Cirrhosis (5–6) | 82(81.2) |
| Unknown | 0(0.0) |
| Bone metastasis |  |
| No | 98(97.0) |
| Yes | 3(3.0) |
| Lung metastasis |  |
| No | 91(90.1) |
| Yes | 10(9.9) |
| Grade |  |
| Ⅰ | 7(6.8) |
| Ⅱ | 45(44.6) |
| Ⅲ | 44(43.6) |
| Ⅳ | 5(5.0) |

Δ: *Others include American Indian, AK Native, Asian and Pacific Islander; LD: Local Destruction; SR: Segmental Resection; LR: Larger Resection; LT: Liver Transplantation; AFP: Alpha Fetoprotein; FS:Fibrosis score.*

**Figure S2** The receiver operating characteristic (ROC) curves of nomogram in external validation set


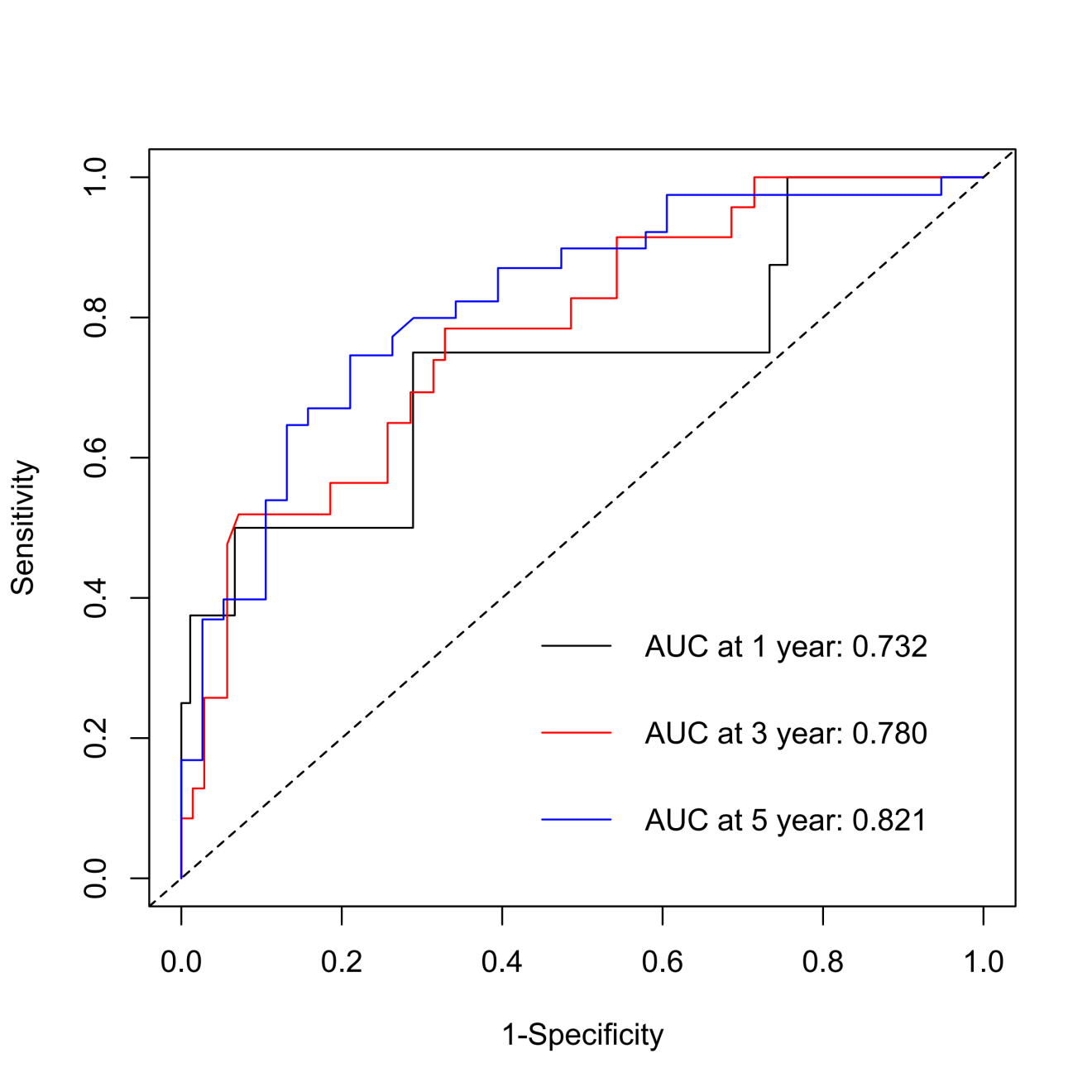


**Figure S3** OS of HCC in training set **(A-B)** and internal validation set **(C-D)**

**
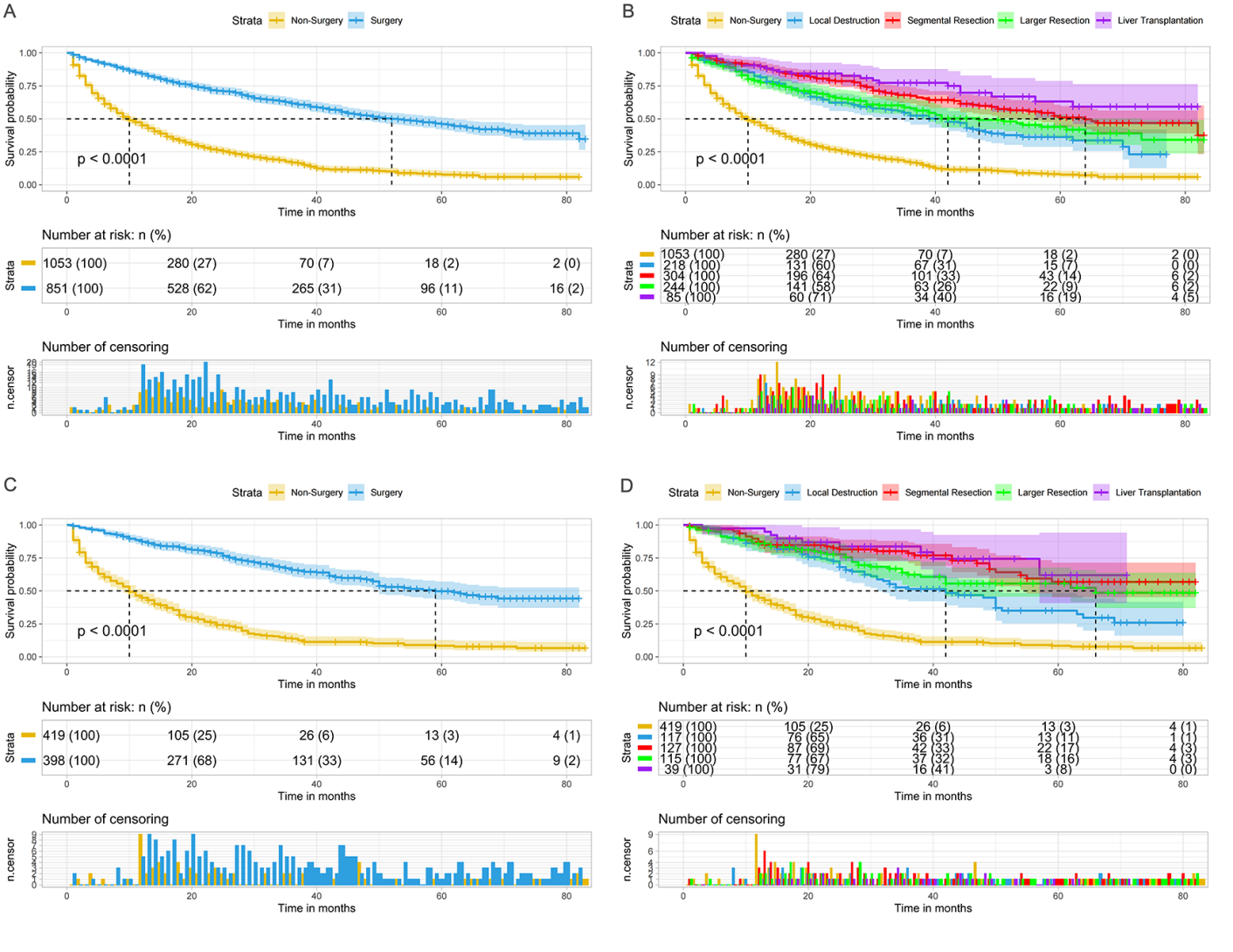
**
